# Clinical and prognosis value of the number of metastatic lymph nodes in patients with papillary thyroid carcinoma

**DOI:** 10.1186/s12893-022-01635-7

**Published:** 2022-06-20

**Authors:** Ling Zhan, Hong-fang Feng, Xi-zi Yu, Ling-rui Li, Jun-long Song, Yi Tu, Jing-ping Yuan, Chuang Chen, Sheng-rong Sun

**Affiliations:** 1 Department of Breast and Thyroid Surgery, Renmin Hospital of Wuhan University, 17th Tiyu Street, Wuhan, Hubei 430060 The People’s Republic of China; 2 Department of Anesthesiology, East Hospital, Renmin Hospital of Wuhan University, Wuhan, Hubei 430060 The People’s Republic of China; 3 Department of Breast Surgery, Thyroid Surgery, Huangshi Central Hospital of Edong Healthcare Group, Affiliated Hospital of Hubei Polytechnic University, Huangshi, 435000 Hubei, The People’s Republic of China; 4 Department of Pathology, Renmin Hospital of Wuhan University, Wuhan, Hubei 430060 The People’s Republic of China

**Keywords:** Papillary thyroid carcinoma (PTC), Number of metastatic lymph nodes (NMLNs), Number of central cervical lymph nodes (CLNs), Number of lateral lymph nodes (LLNs)

## Abstract

**Objective:**

It has been reported that papillary thyroid carcinoma (PTC) patients with lymph node metastasis (LNM) are largely associated with adverse outcomes. The present study aimed to assess the correlation between the number of metastatic lymph nodes (NMLNs) and clinical prognosis in patients with PTC.

**Methods:**

We retrospectively reviewed the medical records of patients with PTC who underwent initial thyroid cancer surgery in Renmin Hospital of Wuhan University between 2017 and 2019. A total of 694 patients with PTC and cervical lymph node dissection as well as a total checked number of lymph nodes ≥ 5 were involved in this study. The clinicopathological characteristics of patients were compared according to NMLNs, the number of central cervical lymph nodes (CLNs) and the number of lateral lymph nodes (LLNs).

**Results:**

NMLNs > 5, CLNs > 5 and LLNs > 5 were 222 (32.0%), 159 (24.3%) and 70 (10.1%) seen in the analyzed samples, respectively. Young patients, patients with larger tumor diameter, bilaterality, multifocality and gross extrathyroidal extension (ETE) were more inclined to NMLNs > 5, CLNs > 5 and LLNs > 5 (*P* < 0.05). It was found that the recurrence-free survival among pN1 patients was significantly discrepant between different groups (NMLNs ≤ 5/5: *P* = 0.001; LLNs ≤ 5/5:* P* < 0.001). In multivariate logistic regression analysis, patients aged < 55 years (OR = 1.917), primary tumor size > 10 mm (OR = 2.131), bilaterality (OR = 1.889) and tumor gross ETE (OR = 2.759) were independent predictors for high prevalence of total NMLNs > 5 (*P* < 0.05). Specially, patients aged < 55 years (OR = 2.864), primary tumor size > 10 mm (OR = 2.006), and tumor gross ETE (OR = 2.520) were independent predictors for high prevalence of CLNs > 5 (*P* < 0.01); Bilaterality (OR = 2.119), CLNs > 5 (OR = 6.733) and tumor gross ETE (OR = 4.737) were independent predictors for high prevalence of LLNs > 5 (*P* < 0.05).

**Conclusions:**

In conclusion, it is evident that NMLNs is related to the invasive clinicopathological features and adverse outcome of patients with PTC which should be correctly evaluated to provide an appropriate guidance for reasonable treatment and careful follow-up.

## Background

Papillary thyroid carcinoma (PTC) is the most common type of thyroid carcinoma (TC). It has a low rate of mortality, even in its advanced stages, and an excellent 10-year overall survival [1]. However, the incidence and recurrence rate of PTC is gradually increasing throughout the world [2, 3]. Further, it has been reported that 66.7% of recurrence incidences of PTC occurred between 5 and 10 years after operation [4, 5]. Notably, when recurrence and distant metastasis occur, the treatment of PTC is usually more compliable and difficult. Several previous studies have reported the involvement of central cervical lymph node metastasis (CLNM) and lateral cervical lymph node metastasis (LLNM) in patients with PTC and 80% of long-term recurrence cases have locoregional cervical lymph nodes metastasis (LNM) (pN1) [6–8]. In addition, several studies have also reported that LNM is a reliable predictor for recurrence [9, 10] and poor prognosis of PTC [7, 11]. However, there are guidelines on the management of PTC proposing different opinions on the management of central cervical lymph nodes (LNs) in different countries and regions [1, 12, 13].

Many researchers [9, 14] have conducted meta-analyses on the risk factors of LNM and found that the risk factors of LNM in PTC patients were inconsistent across different studies. In recent years, more researcher attention is being applied to the features of metastatic LNs, such as the location of metastatic LNs, the number of metastatic lymph nodes (NMLNs), and the maximum diameter of the metastatic LNs and the metastatic LN ratio (mLNR). On the one hand, many scholars have tried to establish a standard for recurrence risk stratification of PTC patients according to the characteristics of metastasis LNs. They found that the risk of recurrence was higher among PTC patients with larger metastatic LNs or higher mLNR [15–18].

As declared in the most recent American Thyroid Association (ATA) risk stratification system, the recurrence rate of pN1 PTC with ≤ 5 LNs involved or > 5 LNs involved is 5% and 20%, respectively [1]. Nevertheless, in the AJCC TNM staging system [19], postoperative LN involvement was only divided into CLNM alone (pN1a) and LLNM (pN1b). Therefore, many scholars have suggested addition of the features of metastatic LNs into the current American Joint Committee on Cancer (AJCC) staging system [16, 18]. On the other hand, some investigators have also tried to analyze the clinical response and different characteristics of metastatic LNs of patients with PTC. For instance, a previous study conducted by Gao et al. [20] found that excellent therapeutic effect was mainly among patients with lower mLNR and NMLNs.

Determination of patients with high-risk and administration of appropriate treatment are essential in the clinical treatment and management of PTC. The clinical management of cervical LNs for patients with PTC is still controversial because there is no requirement for a minimum number of lymph nodes to be sampled [18]. This retrospective study explored the relationship between the features of LNM and NMLNs as well as the outcome in patients with PTC and hence aimed to provide substantial evidence for the medical practice of PTC with LNM.

## Methods and patients

### Selection of patients

**Fig. 1:**
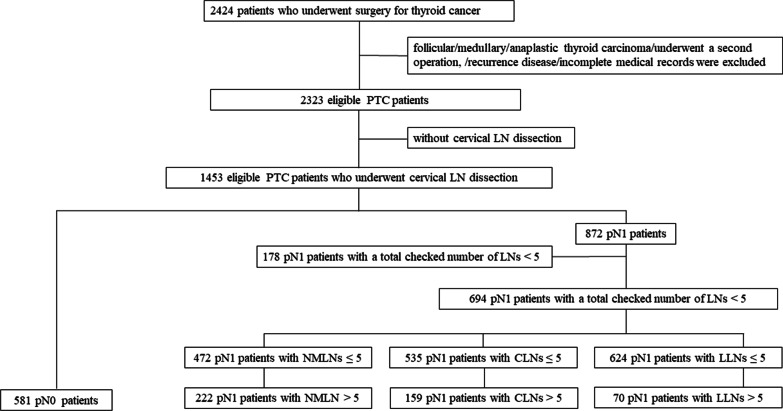
Diagram shows the process of selecting qualified patients for analysis

Retrospective collection of data was obtained for 2424 patients who underwent surgery for TC from 2017 to 2019 at the Department of Breast and Thyroid Surgery of the Renmin Hospital of Wuhan University (Wuhan, Hubei, the People's Republic of China). The process of selecting the qualified patients for analysis was as shown in Fig. 1. Patients who had follicular, medullary, or anaplastic thyroid carcinoma, or underwent a second operation, or had recurrence disease, or had incomplete medical records were excluded. Further, patients who did not undergo central cervical lymph node dissection (CLND) were also excluded in the present study [1].

The participants were categorized into two groups according to the stage of lymph node (LN) and then they were comparatively analyzed. The clinicopathological characteristics of patients with pN1 were further compared based on NMLNs. A few articles [16–18] have found that a greater NMLNs may be associated with lower survival. Similarly, patients with NMLNs ≥ 5 and NMLNs < 5 were divided into intermediate-risk and low-risk according to the improved risk stratification of recent ATA guideline [1].

Nevertheless, in the AJCC TNM staging system [19], postoperative LN involvement was only divided into CLNM alone (pN1a) and LLNM (pN1b). In addition, the number of LNs sampled and the NMLNs are still no relevant cut-off value till now. The current study aimed to evaluate the clinical and prognosis significance of NMLNs ≥ 5 among pN1 patients. It was noted that there were great differences in cervical LNM of patients with PTC and the surgical treatment of LNs for patients with PTC among different hospitals is still different and depends on the judgment of surgeon on the procedural safety [6, 12, 13, 21].

According to the total number of LNs checked, data for 694 eligible pN1 patients who had a total checked number of LNs ≥ 5 was retrieved because the real NMLNs could not be elucidated for those patients with a total checked number of LNs < 5. The reasons are as follows: on the one hand, the NMLNs among the patients were maybe less than 5, but on the other hand, it could also be possible that the final calculated NMLNs were less than 5 due to an insufficient checked number of LNs. The present study aimed to understand the clinical and prognostic significance of NMLNs ≥ 5. Therefore, the patients had to be excluded when comparing the pN1 patients with NMLNs ≥ 5 and NMLNs < 5. Institutional Ethics was approved by the Review Committee of the Renmin Hospital of Wuhan University approved the study (No. 2042019kf0229). All the patients signed written informed consent.

### Surgical treatment

The selected patients underwent lobectomy or total thyroidectomy (includes isthmus) based on the criteria outlined in the 2015 ATA manual, with prophylactic ipsilateral CLND therapeutic as a standard procedure in the hospital for participants of the current study. Modified radical lateral neck dissection (MND) was simultaneously performed for patients with a sign of LLNM in preoperative cervical ultrasonography (US) and then confirmed as LLNM by fine-needle aspiration pathology [1]. The CLND extends superiorly to the hyoid bone, inferiorly to the innominate vein, laterally to the carotid sheath, and dorsally to the prevertebral fascia. The improved MND is defined as the complete removal of level II to level IV lateral neck LNs. The levels I and V dissections were not performed if there was no evidence for metastasis.

### Pathological examination

All participants were routinely examined preoperatively through cervical US and postoperatively through pathological examination. Pathological findings were obtained from the Department of Pathology, Renmin Hospital of Wuhan University. The report evaluated the maximum diameter of the primary tumor, unilaterality or bilaterality, number of lesions, the presence or absence of ETE, the location of metastatic LNs, and the NMLNs, tumor node metastasis (TNM) staging, BRAF status, as well as the presence of Hashimoto’s thyroiditis (HT).

The presence of two or more tumor foci of PTC regardless of unilaterality or bilaterality was defined as multifocality. According to the degree of invasion, the histological ETE was divided into ETE and no ETE. Further, the regional metastatic LNs can be divided into no cervical LNM (pN0), CLNM alone (pN1a), and LLNM (pN1b) based on postoperative pathology. The present study was focused on the characteristics of metastatic LNs including the location and NMLNs. Pathological staging of tumors was adopted from the eighth edition of AJCC TNM staging system [19], which shows no difference based on the difference between pN1a and pN1b. Because medical insurance did not cover the cost of genetic testing, consequently, only a small number of patients (173 cases) were tested with BRAF^V600E^ mutation. Furthermore, the hospital lacks information on several other mutation gene mutations, such as p53 and TERT.

### Follow-up

Serum-free triiodothyronine (FT3), serum-free thyroxine (FT4), and serum thyroid-stimulating hormone (TSH) of all the participating patients were checked one month after the operation to determine whether there was hypothyroidism. The FT3, FT4, and TSH can be defined as three biochemical tests for thyroid function. Consequently, biochemical tests for thyroid function and level of thyroglobulin (TG) and check for the cervical US were conducted every three months. An abnormal increase of TG or metastasis of previous thyroid and LNs in the cervical US were evaluated as signs for recurrence in the present study. For patients who had a recurrence of PTC, it is noted that surgery should be performed to remove thyroid lesions or LNs in the affected area. The follow-up time was from the operation date to the recurrence or the last follow-up months (July 1st, 2020).

### Statistical analysis

The data were analyzed using SPSS 25.0 software program (IBM Corp., Armonk, NY, USA). The continuous variables were described using mean ± standard deviation (M ± SD) and median. The significant difference between the various groups was analyzed using Student's t-test or the rank test. The classified variables were expressed as the number of cases in percentage. The chi-square test or Fisher’s exact test was performed for categorical variables. Log rank tests and Kaplan Meier method were employed to compare the recurrence-free survival (RFS). Univariate logistic regression analysis was applied to calculate odds ratios (ORs) and the 95% confidence intervals (CIs) for NMLNs > 5, including CLNs > 5 and LLNs > 5. Variables with a *P*-value < 0.05 were included in the multivariate logistic regression analysis to determine the association between clinicopathological characteristics and NMLNs > 5. All tests were two-sided and *P*-value < 0.05 was indicated statistical significance.

## Results

### Baseline clinicopathological characteristics of patients with PTC

The baseline clinicopathological characteristics of 1453 patients with PTC were as shown in Table 1. Results of the present study revealed that with a median age of 46 years old and a median primary tumor size of 8.0 mm, the ratio of females and males of all eligible patients was 74.7% and 25.3%, respectively. A total of 863 (42.9%) patients were diagnosed as papillary thyroid microcarcinoma (PTMC) by pathology. Further, the pN0 and pN1 were found in 581 (40.0%) and 872 (60.0%) participants, respectively. The total checked number of LNs ≥ 5 or < 5 among the pN1 patients were 694 (79.6%) and 178 (20.4%), respectively. Further analysis showed that NMLNs > 5, CLNs > 5 and LLNs > 5 were 222 (32.0%), 159 (24.3%) and 70 (10.1%) seen among pN1 patients with a total checked number of LNs ≥ 5, respectively. Bilaterality, multifocality and ETE was confirmed in 28.0%, 36.5%, 72.0% of patients. The proportion of TNM stage I and II was 89.7% and 10.3%. There were 143 and 196 cases of BRAF^V600E^ mutation and HT, respectively. In addition, the median time interval follow-up was 22 months (range from 4 and 42 months). Moreover, five cases with a recurrence of cervical LNs were identified across all patients whereas all of them were relieved after a thorough dissection of the metastatic LNs and radioiodine ablation (RAI) treatment. Five cases with a recurrence of cervical LNs were identified across all patients.

**Table 1:** Baseline clinicopathological characteristics of patients with PTC

Variates	Value
Cases of patients	1453
Age, median (range)	44.8 ± 11.9, 46 (15–88)
Sex, male/female, number (%)	368/1085 (25.3/74.7)
Primary tumor size, median (mm)	11.1 ± 7.2, 8.0 (0.1–55.0)
PTMC/non-PTMC, number (%)	863/590 (59.4/40.6)
LN involvement, pN0/ pN1a/ pN1b, number (%)	581/698/174 (40.0/48.0/12.0)
total checked number of LNs ≥ 5/ < 5 among pN1patients	694/178(79.6/20.4)
NMLNs among pN1patients with a total checked number of LNs ≥ 5, NMLNs ≤ 5/ NMLNs > 5, number (%)	472/222 (68.0/32.0)
CLNs among pN1patients with a total checked number of LNs ≥ 5, CLNs ≤ 5/ > 5, number (%)	535/159 (75.7/24.3)
LLNs among pN1patients with a total checked number of LNs ≥ 5, LLNs ≤ 5/ > 5, number (%)	624/70(89.9/10.1)
Bilaterality, number (%)	407 (28.0)
Multifocality, number (%)	531 (36.5)
ETE /without ETE, number (%)	1046/407 (72.0/28.0)
TNM stage, I/II, number (%)	1304/149 (89.7%/10.3%)
BRAF^*V600E*^ mutation, positive/negative/unknown, number (%)	143/30/1280 (9.8/2.1/88.1)
HT, number (%)	196 (13.5)
Duration of follow up (months)	4–42 (22)

*NMLNs* number of metastatic lymph nodes, *CLNs* number of central cervical lymph nodes metastasis, *LLNs* number of lateral lymph nodes metastasis, *ETE* extrathyroidal extension, *TNM stage* tumor node metastasis stage, *HT* Hashimoto’s thyroiditis

### Clinicopathological characteristics of pN1 patients based on NMLNs

According to the ATA guidelines of 2015, N1 PTC with ≤ 5 LNs involved or > 5 LNs involved had different recurrence risk. To verify the clinical significance of NMLNs > 5 among pN1 patients, a comparison of clinicopathological characteristics of pN1 patients was based on NMLNs ≤ 5 or > 5. Before that, 178 pN1 patients were ruled out with a total checked number of LNs < 5 which real NMLNs could not be established.

As shown in Table 2, among pN1 patients with a total checked number of LNs ≥ 5, NMLNs ≤ 5 and NMLNs > 5 was evident in 472 (68.0%) and 222 (32.0%), respectively. Patients with NMLNs > 5 were significantly younger as compared with those with NMLNs ≤ 5 (37.0 years vs 44.0 years, *P* < 0.001). Although patients with NMLNs > 5 were more common among the males, it was found that there was no statistical significance (33.3% vs 27.1%, *P* = 0.093). The NMLNs > 5 was significantly associated with larger tumour diameter (14.0 mm vs 9.0 mm, *P* < 0.001), bilaterality (49.5% vs 30.1%, *P* < 0.001), multiplicity (54.1% vs 39.2%, *P* < 0.001), and ETE (92.3% vs 76.3%, *P* < 0.001), as compared with NMLNs ≤ 5. Further analysis showed that non-PTMC patients were prone to NMLNs > 5 compare to PTMC patients (68.9% vs 33.1%, *P* < 0.001). However, there was a negative correlation between the advanced TNM stage (11.3% vs 17.6%, *P* < 0.05) in NMLNs > 5 as compared with NMLNs ≤ 5. However, HT or a positive BRAF^*V600E*^ mutation was not associated with NMLNs.

**Table 2:** Clinicopathological characteristics of pN1 patients based on NMLNs

Clinicopathological characteristics	NMLNs ≤ 5 n = 472	NMLNs > 5 n = 222	Total n = 694	*P* value
Age (years)				
Mean	44.0	37.0		< 0.001*
< 55	389/472 (82.4)	196/222 (88.3)	585	0.047*
≥ 55	83/472 (17.6)	26/222 (11.7)	109	
Sex				0.093
Female	344/472 (72.9)	148/222 (66.7)	492	
Male	128/472 (27.1)	74/222 (33.3)	202	
Tumor size (mm)				
Mean	9.0	14.0		< 0.001*
≤ 10	260/472 (55.1)	69/222 (31.1)	329	< 0.001*
10–20	164/472 (34.7)	103/222 (46.4)	267	
20–40	40/472 (8.5)	43/222 (19.4)	83	
> 40	8/472 (1.7)	7/222 (3.1)	15	
Bilaterality				< 0.001*
Present	142/472 (30.1)	110/222 (49.5)	252	
Absent	330/472 (69.9)	112/222 (50.5)	442	
Multifocality				< 0.001*
Present	185/472 (39.2)	120/222 (54.1)	305	
Absent	287/472 (60.8)	102/222 (45.9)	389	
ETE				< 0.001*
Present	360/472 (76.3)	205/222 (92.3)	565	
Absent	112/472 (23.7)	17/222 (7.7)	129	
TNM stage				0.032*
I	389/472 (82.4)	197/222 (88.7)	586	
II	83/472 (17.6)	25/222 (11.3)	108	
HT				0.935
Present	67/472 (14.2)	31/222 (14.0)	98	
Absent	405/472 (85.8)	191/222 (86.0)	596	
BRAF ^*V600E*^ mutation				0.166
Positive	46/55 (83.6)	22/31 (71.0)	68	
Negative	9/55 (16.4)	9/31 (29.0)	18	

*NMLNs* number of metastatic lymph nodes, *ETE* extrathyroidal extension, *TNM stage* tumor node metastasis stage, *HT* Hashimoto’s thyroiditis

*Statistically significant difference

### Clinicopathological characteristics of pN1 patients based on CLNs and LLNs

The clinicopathological characteristics of pN1 patients based on CLNs and LLNs were as shown in Table 3. It was found that CLNs > 5 and LLNs > 5 were evident in 159 (24.3%) and 70 (10.1%) among pN1 patients with a total checked number of LNs ≥ 5, CLNs > 5 and LLNs > 5 were evident in 159 (24.3%) and 70 (10.1%), respectively. The patients with CLNs > 5 were significantly younger as compared with those with CLNs ≤ 5 (34.0 years vs 44.0 years, *P* < 0.001). CLNs > 5 and LLNs > 5 was significantly associated with larger tumour diameter (14.0 mm vs 10.0 mm, *P* < 0.001; 15.0 mm vs 11.0 mm, *P* < 0.001;), bilaterality (49.7% vs 32.3%, *P* < 0.001; 58.6% vs 33.8%, *P* < 0.001), multiplicity (56.0% vs 40.4%, *P* < 0.01; 58.6% vs 42.3%, *P* < 0.001), and ETE (92.5% vs 78.1%, *P* < 0.001; 97.1% vs 79.6%, *P* < 0.001), as compared with CLNs ≤ 5 and LLNs ≤ 5, respectively. Further analysis showed that non-PTMC were prone to CLNs > 5 (66.7% vs 33.3%, *P* < 0.001) and LLNs > 5 (64.3% vs 35.7%, *P* < 0.001) as compare with PTMC. Besides, CLNs > 5 were more relevent with LLNs > 5 (65.7% vs 34.3%, *P* < 0.001). However, HT or a positive BRAF^*V600E*^ mutation was not associated with CLNs and LLNs.

**Table 3:** Clinicopathological characteristics of pN1 patients based on CLNs and LLNs

Clinicopathological characteristics	Total n = 694	CLNs ≤ 5 n = 535	CLNs > 5 n = 159	*P* value	LLNs ≤ 5 n = 624	LLNs > 5 n = 70	*P* value
Age (years)							
Mean		44.0	34.0	< 0.001*	42.0	37.5	0.040*
< 55	585	439/535 (82.1)	146/159 (91.8)	0.003*	523/624 (83.8)	62/70 (88.6)	0.300
≥ 55	109	96/535 (17.9)	13/159 (8.2)		83/624 (17.6)	26/222 (11.7)	
Sex				0.053			0.314
Female	492	389/535 (72.7)	103/159 (64.8)		446/624 (71.5)	46/70 (65.7)	
Male	202	146/535 (27.3)	56/159 (35.2)		178/624 (28.5)	24/70 (34.3)	
Tumor size (mm)							
Mean		10.0	14.0	< 0.001*	11.0	15.0	< 0.001*
≤ 10	329	276/535 (51.6)	53/159 (33.3)	< 0.001*	304/624 (48.7)	25/70 (35.7)	0.001*
10–20	267	201/535 (37.6)	66/159 (41.5)		242/624 (38.8)	25/70 (35.7)	
20–40	83	48/535 (9.0)	35/159 (22.0)		67/624 (10.7)	16/70 (22.9)	
> 40	15	10/535 (1.9)	5/159 (3.1)		11/624 (1.8)	4/70 (5.7)	
Bilaterality				< 0.001*			< 0.001*
Present	252	173/535 (32.3)	79/159 (49.7)		211/624 (33.8)	41/70 (58.6)	
Absent	442	362/535 (67.7)	80/159 (50.3)		423/624 (66.2)	29/70 (41.4)	
Multifocality				0.001*			< 0.001*
Present	305	216/535 (40.4)	89/159 (56.0)		264/624 (42.3)	41/70 (58.6)	
Absent	389	319/535 (59.6)	70/159 (44.0)		360/624 (57.7)	29/70 (41.4)	
CLNs				^−^			< 0.001*
≤ 5	535	–	–		511/624 (81.9)	24/70 (34.3)	
> 5	159	–	–		113/624 (18.1)	46/70 (65.7)	
ETE				< 0.001*			< 0.001*
Present	565	418/535 (78.1)	147/159 (92.5)		497/624 (79.6)	68/70 (97.1)	
Absent	129	117/535 (21.9)	12/70 (7.5)		127/624 (20.4)	2/70 (2.9)	
TNM stage				0.001*			0.314
I	586	439/535 (82.1)	147/159 (92.5)		524/624 (84.0)	62/70 (88.6)	
II	108	96/535 (17.9)	12/159 (7.5)		100/624 (16.0)	8/70 (11.4)	
HT				0.509			0.686
Present	98	73/535 (13.6)	25/159 (15.7)		87/624 (13.9)	11/70 (15.7)	
Absent	596	462/535 (86.4)	134/159 (84.3)		537/624 (86.1)	59/70 (84.3)	
BRAF ^*V600E*^ mutation				0.234			0.479
Positive	68	52/63 (82.5)	16/23 (69.6)		58/72 (80.6)	10/14 (71.4)	
Negative	18	11/63 (17.5)	7/23 (30.4)		14/72 (19.4)	4/14 (28.6)	

*CLNs* number of central cervical lymph nodes, *LLNs* number of lateral lymph nodes, *ETE* extrathyroidal extension, *TNM stage* tumor node metastasis stage, *HT* Hashimoto’s thyroiditis

*Statistically significant difference

### Correlation between clinicopathological characteristics of pN1 patients and the NMLNs > 5

The association between clinicopathological characteristics and the NMLNs > 5 was analyzed in pN1 patients. As depicted in Table 4, the patients aged < 55 years old (OR = 1.917, *P* < 0.05), primary tumor size > 10 mm (OR = 2.131, *P* < 0.001), bilaterality (OR = 1.889,* P* < 0.01) and tumor gross ETE (OR = 2.759, *P* < 0.001) were independent predictors for the high prevalence of NMLNs > 5.

**Table 4:** Correlation between clinicopathological characteristics of pN1 patients and the NMLNs > 5

Clinicopathological characteristics	Univariate analysis		Multivariate analysis	
	OR (95%CI)	P value	OR (95%CI)	P value
Age (years old)		0.013*		0.010*
≥ 55	1 (reference)		1 (reference)	
< 55	1.856 (1.142–3.016)		1.917 (1.167–3.150)	
Gender		0.063	NA	NA
Female	1 (reference)			
Male	1.398 (0.982–1.990)			
Primary tumor size (mm)		< 0.001*		< 0.001*
≤ 10	1 (reference)		1 (reference)	
> 10	2.568 (1.832–3.600)		2.131 (1.497–3.035)	
Bilaterality		< 0.001*		0.005*
No	1 (reference)		1 (reference)	
Yes	2.298 (1.653–3.194)		1.889 (1.208–2.954)	
Multifocality		< 0.001*		0.612
No	1 (reference)		1 (reference)	
Yes	1.833 (1.327–2.531)		1.121 (0.722–1.741)	
ETE		< 0.001*		< 0.001*
No	1 (reference)		1 (reference)	
Yes	3.817 (2.225–6.547)		2.759 (1.577–4.828)	

*NMLNs* number of metastatic lymph nodes, *ETE* extrathyroidal extension

*Statistically significant difference

### Correlation between clinicopathological characteristics of pN1 patients and CLNs > 5

The association between clinicopathological characteristics and CLNs > 5 was analyzed in pN1 patients. As depicted in Table 5, the patients aged < 55 years old (OR = 2.864, *P* < 0.01), primary tumor size > 10 mm (OR = 2.006, *P* < 0.01), and tumor gross ETE (OR = 2.520, *P* < 0.01) were independent predictors for the high prevalence of CLNs > 5.

**Table 5:** Correlation between clinicopathological characteristics of pN1 patients and the CLNs > 5

Clinicopathological characteristics	Univariate analysis		Multivariate analysis	
	OR (95%CI)	P value	OR (95%CI)	P value
Age (years old)		0.004*		0.001*
≥ 55	1 (reference)		1 (reference)	
< 55	2.448 (1.330–4.504)		2.864 (1.532–5.353)	
Gender		0.058	NA	NA
Female	1 (reference)			
Male	1.443 (0.987–2.109)			
Primary tumor size (mm)		< 0.001*		0.001*
≤ 10	1 (reference)		1 (reference)	
> 10	1.646 (1.374–1.972)		2.006 (1.352–2.976)	
Bilaterality		< 0.001*		0.056
No	1 (reference)		1 (reference)	
Yes	2.083 (1.451–2.988)		1.611 (0.987–2.628)	
Multifocality		0.001*		0.308
No	1 (reference)		1 (reference)	
Yes	1.888 (1.319–2.703)		1.287 (0.792–2.092)	
ETE		< 0.001*		0.005*
No	1 (reference)		1 (reference)	
Yes	3.499 (1.873–6.536)		2.520 (1.322–4.806)	

*CLNs* number of central cervical lymph nodes, *ETE* extrathyroidal extension

*Statistically significant difference

### Correlation between clinicopathological characteristics of pN1 patients and LLNs > 5

The association between clinicopathological characteristics and the LLNs > 5 was analyzed in pN1 patients. As depicted in Table 6, bilaterality (OR = 2.119, *P* < 0.05), CLNs > 5 (OR = 6.733, *P* < 0.001), and tumor gross ETE (OR = 4.737, *P* < 0.05) were independent predictors for the high prevalence of LLNs > 5.

**Table 6:** Correlation between clinicopathological characteristics of pN1 patients and the LLNs > 5

Clinicopathological characteristics	Univariate analysis		Multivariate analysis	
	OR (95%CI)	P value	OR (95%CI)	P value
Age (years old)		0.308	NA	NA
≥ 55	1 (reference)			
< 55	1.490(0.692–3.208)			
Gender		0.323	NA	NA
Female	1 (reference)			
Male	0.768(0.455–1.296)			
Primary tumor size (mm)		< 0.001*		0.086
≤ 10	1 (reference)		1 (reference)	
> 10	2.745(1.570–4.800)		1.694(0.928–3.094)	
Bilaterality		< 0.001*		0.043*
No	1 (reference)		1 (reference)	
Yes	2.779(1.678–4.601)		2.119(1.024–4.387)	
Multifocality		0.010*		0.664
No	1 (reference)		1 (reference)	
Yes	1.932(1.170–3.192)		0.850(0.409–1.768)	
CLNs		< 0.001*		< 0.001*
≤ 5	1 (reference)		1 (reference)	
> 5	8.601(5.035–14.692)		6.733(3.891–11.654)	
ETE		0.003*		0.036*
No	1 (reference)		1 (reference)	
Yes	8.799(2.127–36.405)		4.737(1.106–20.285)	

*CLNs* number of central cervical lymph nodes, *LLNs* number of lateral lymph nodes, *ETE* extrathyroidal extension

*Statistically significant difference

### Recurrence-free survival based on LN stage and NMLNs

**Fig. 2:**
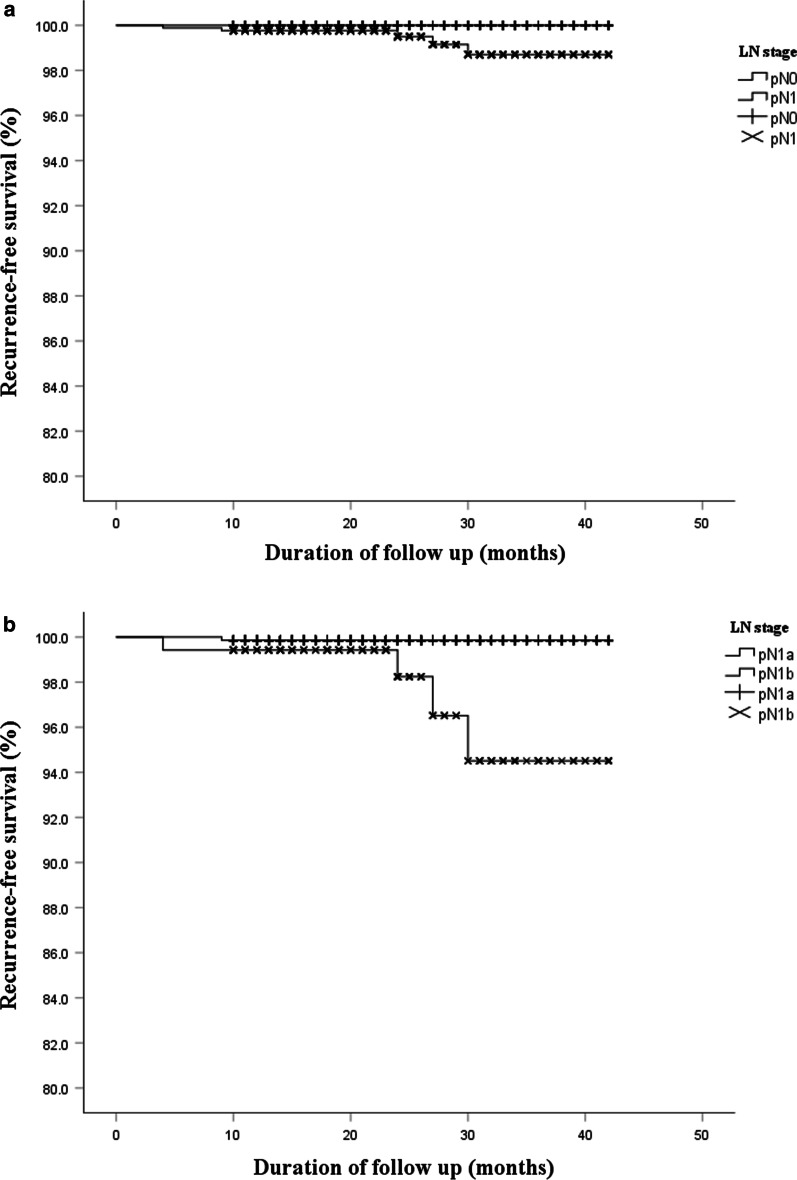
RFS according to the LN stage by Kaplan–Meier curves. **a** There was no significant difference in RFS between pN0 and pN1 (100.0% vs 99.4%, *P* = 0.05). **b** These showed poorer RFS in the pN1b than pN1a (97.7% vs 99.9%, *P* = 0.001);

**Fig. 3:**
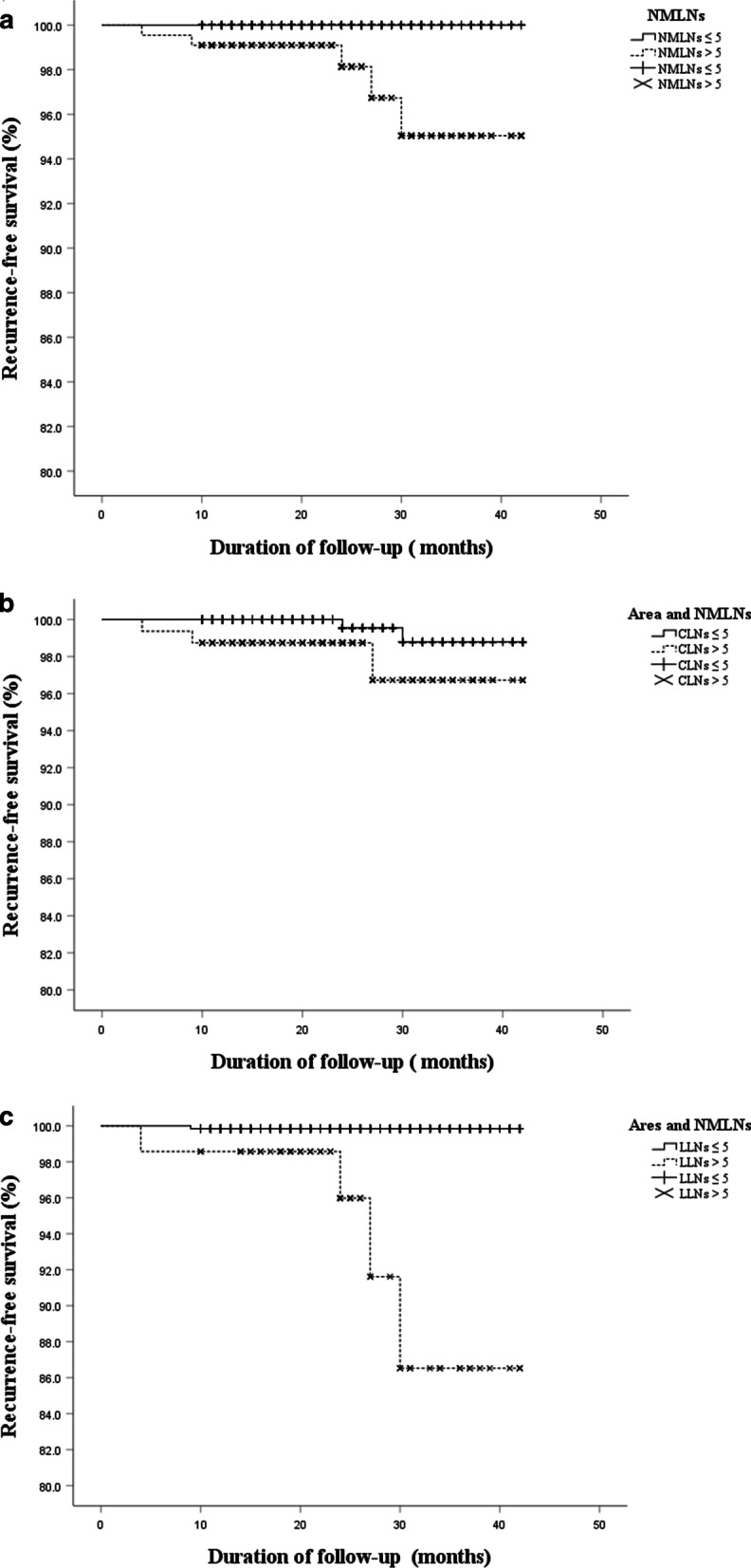
RFS according to the NMLNs by Kaplan–Meier curves. **a** These showed poorer RFS in the NMLNs > 5 than NMLNs ≤ 5 (97.7% vs 100.0%, *P* = 0.001). **b** There was no significant difference in RFS between CLNs ≤ 5 and CLNs > 5 (99.6% vs 98.1%, *P* = 0.051). **c** These showed poorer RFS in the LLNs > 5 than LLNs ≤ 5 (94.3% vs 99.8%, *P* < 0.001)

During the median follow-up period of 22 months (range between 4 and 42 months), five cases of disease recurrence were identified across all patients. As depicted in Table 7, there existed a significant 3-year RFS discrepancy in different groups. pN1b reduced recurrence-free survival **(**RFS) (pN1a / pN1b: *P* < 0.01) (Fig. 2a, b). RFS among pN1 patients was significantly different between different groups (NMLNs ≤ 5/5: *P* < 0.001; LLNs ≤ 5/5:* P* < 0.001) (Fig. 3a–c).

**Table 7:** Correlation between LNM, NMLNs and recurrence

Variates	No recurrence	Recurrence	Total	*P* value
LN stage				0.050
pN0	581 (100.0)	0 (0.0)	581	
pN1	867 (99.4)	5 (0.6)	872	
LN stage				0.001*
pN1a	697 (99.9)	1 (0.1)	698	
pN1b	173 (97.7)	4 (2.3)	174	
NMLNs				0.001*
≤ 5	472 (100.0)	0 (0.0)	472	
> 5	217 (97.7)	5 (2.3)	222	
Area and NMLNs				
CLNs ≤ 5	533 (99.6)	2 (0.4)	535	0.051
CLNs > 5	156 (98.1)	1 (1.9)	159	
LLNs ≤ 5	623 (99.8)	1 (0.2)	624	< 0.001*
LLNs > 5	66 (94.3)	4 (5.7)	70	

*NMLNs* number of metastatic lymph nodes, *CLNs* number of central cervical lymph nodes, *LLNs* number of lateral lymph nodes, *ETE* extrathyroidal extension, *TNM stage* tumor node metastasis stage, *HT* Hashimoto’s thyroiditis

## Discussion

Although a good prognostic value of PTC was reported, a rate of recurrence, long-distance metastasis, and disease-specific mortality among patients with PTC who were diagnosed as LNM were also evident [2, 9, 22, 23]. Therefore, knowledge of the characteristic of patients with LNM and the predictors for LNM in patients with PTC is highly required. The purpose of this study was to evaluate the influence of the location of metastatic LNs and NMLNs on prognosis of patients with PTC. Results of the present study found that pN1 patients having aggressive characteristics, such as a larger primary tumor size, the presence of grossly ETE, were related to a greater NMLNs. Moreover, it was also found that RFS among pN1 patients was significantly discrepant between different groups.

Many previous studies have reported that young people are more likely to have LNM [24], the data obtained from the study revealed that patients aged < 55 years old were more prone to NMLNs > 5, especially CLNs > 5. Besides, aged < 55 years old was an independent predictor for the high prevalence of NMLNs > 5 and CLNs > 5. Whether prophylactic CLND should be recommended during thyroidectomy for patients younger than 55 years old is still controversial. On one hand, prophylactic CLND may lead complications that might significantly influence the quality of life [1]. On the other hand, patients older than 45 years old is an independent risk factor for recurrence [25]. Therefore, a balance between the risks and benefits of prophylactic CLND deserves further discussion. We suggest that patients aged between 45 and 55 years old should undergo CLND at the same time of thyroid surgery to reduce the risk of recurrence. In our study, ratio of female /male ratio was 1:3, which was consistent with previous studies [2]. Nevertheless, no relationship was observed between male patients and NMLNs > 5, including CLNs > 5 and LLNs > 5, which were not consistent with the finding previously reported by Sheng et al. [24], who found that male predict an increased number of CLNM (n ≥ 5) in patients with PTMC.

A characteristic relationship between metastatic LNs and the original PTC lesion was also detected. The PTMC, with a diameter ≤ 10 mm, has a better prognosis than PTC [1]. Although investigators alarmed that PTMC might occur in LNM before diagnosis [26], pathological data from patients in our study confirmed that non-PTMC was an independent risk factor for NMLNs > 5, especially for CLNs > 5. This could imply that patients with PTMC were at a low-risk of NMLNs > 5 and CLNs > 5. Besides surgery, active surveillance could also be an alternative approach for patients with PTMC [1]. Hence, a larger tumor size could be a risk factor for worse LN status, which should be a higher concern for the clinicians. Several studies have concluded that patients with LNM are likely associated with bilateral cancer and multifocality [11, 27], it was found that bilaterality was a high-risk factor for NMLNs > 5 and LLNs > 5 in the present study. The determination of the correlation between LN status and ETE is also critical. In our study, NMLNs > 5 was associated with ETE, which was in agreement with several other reports of previous studies [11, 28]. Furthermore, tumor gross ETE was the risk factor for the high prevalence NMLNs > 5, including CLNs > 5 and LLNs > 5. Previous evidence showed that ETE had a severe influence on the prognosis of patients and high prevalence in the aggressive types of thyroid carcinoma, which cannot be overlooked [19, 29]. According to a study conducted by Li et al. [29], the risk of LN involvement in patients without ETE was similar to that of patients with ETE into perithyroidal tissue but lower than those with T3b and ETE invading beyond the strap muscles. Unfortunately, we did not study the relationship between LNM and different extent of ETE.

Numerous works of literature have reported an association of HT with less CLNM [11, 24, 30]. In this study, coexisting HT was not associated with NMLNs > 5, which was found only in 158 patients, hence the results were not sufficient for a substantive conclusion. The presence of BRAF^*V600E*^ mutation, common in PTC, was closely related to invasive long-term outcomes of PTC, such as higher disease-specific mortality and a shorter RFS [31–33]. A previous study proposed a correlation between LN status and BRAF cytology [34, 35], whereas our data demonstrated BRAF^*V600E*^ mutation was not relevant to NMLNs > 5. It is important to emphasize that patients with the identification of BRAF^*V600E*^ mutation may need more aggressive treatment when there is evidence of LNM, but not if there is no any evidence of LNM [19]. Whether the NMLNs will affect treatment decisions of pN1 patients with a BRAF^*V600E*^ mutation is also worthy of further discussion. Prospective studies with a large sample are needed to elucidate the relationship between BRAF^*V600E*^ mutation and metastatic LNs to establish the appropriate operational and follow-up strategy for the patients with LNM.

Results of the present study found that pN1 patients with CLN > 5 were associated with advanced tumor stage but not reduce 3-year RFS. On the contrary, pN1 patients with LLNs > 5 were associated with a lower RFS. Of course, there is need for a long-time follow-up to testify this phenomenon. The information obtained from the clinicopathological risk factors, related to NMLNs > 5, should be applied to guide any treatment decisions. LNM is one of the most important factors for lobectomy or total thyroidectomy [1]. It was suggested that lobectomy or total thyroidectomy should be determined in combination with the tumor diameter, LN status, the presence or absence of ETE and etc. Total thyroidectomy is feasible for patients with a diameter ≥ 40 mm ETE and LLNM. It was also emphasized that there should be an intense focus on necessity of CLND for all PTC patients by preoperative evaluation of cervical LN status. Although there is no significant reduction in RFS among patients with CLN > 5, it is an independent risk factor for LLN > 5, which reduces RFS. Consequently, we proposed to perform a selective application of prophylactic CLND for patients with a risk of CLNs > 5 and therapeutic CLND for patients with a risk of LLNs > 5. In addition, thyroid be totally removed and therapeutic CLND as well as MND be simultaneously performed among patients with LLNs > 5. A regular follow-up including cervical US and thyroid function test should also be taken for the high-risk population with NMLNs > 5, especially for LLN > 5. The ATA guidelines recommend RAI for high-risk and some moderate-risk patients, but not for low-risk patients [1]. We suggested that pN1b patients with total thyroidectomy should accept a subsequent RAI treatment, which completely remove tumor and residual thyroid tissue to prevent the recurrence and distant metastasis by promoting sufficient I^131^ to enter the residual thyroid tissue and metastatic focus [36, 37]. However, RAI should only be employed in patients with a total thyroidectomy because I^131^ can damage normal thyroid tissue [13]. It may be necessary to evaluate NMLNs and estimate the effect of RAI treatment in the future. Next, A certain time and dose levothyroxine should be given as an alternative postoperative therapy to achieve the goal of individualized and dynamic inhibition of TSH as well as reduce the possibility of tumor recurrence [38]. To conclude, the features of metastatic LNs should be cautiously considered in the postoperative risk stratification, guiding treatment decision-making and follow-up practices.

The study had some limitations. Firstly, it was inevitable to avoid the disadvantages of a single-center and retrospective study. Secondly, the clinicopathological characteristics of patients with different sizes of metastatic LNs were not evaluated due to lack of information on the size of metastatic LNs. Thirdly, the lack of data on 10-years follow-up of disease recurrence and survival prevented the evaluation of the relationship between LN status and recurrence as well as survival.

However, this article is still worthy of reference, especially in the analysis of the characteristics of metastatic LNs in patients with PTC. A larger sample size was enrolled and only patients with PTC who had a total checked number of LNs ≥ 5 were retrieved so that the data made difference to assessing the influence of CLN > 5 and LLN > 5 on the clinicopathological characteristics of patients with PTC. In the future, multiple-center and long-term prospective studies are proposed to clarify the correlation between LN status and various clinicopathological parameters in patients with PTC as well as evaluate the influence of LNM on prognosis.

## Conclusions

Overall, our findings showed that CLNs > 5 was more common in patients with age < 55 years old, largely tumor diameter and tumor gross ETE. LLNs > 5 was more common in patients with age < 55 years old, bilaterality, CLNs > 5, tumor gross ETE and more inclined to have a reduced RFS. However, there is need for sufficient clinical data and molecular biology theory to support the described argument. Further studies on the characteristics of LNM will be essential for prognostic judgment and decision-making in clinical practices to improve the health of patients.

## Data Availability

The datasets used or analyzed during the current study are available from the corresponding author on reasonable request.

## Abbreviations

PTC: Papillary thyroid carcinomas
LNs: Lymph nodes
LNM: Lymph node metastasis
CLNs: Number of central cervical lymph nodes
LLNs: Number of lateral lymph nodes
CLND: Central cervical lymph node dissection
MND: Modified radical lateral neck dissection
NMLNs: Number of metastatic lymph nodes
CLNM: Central cervical lymph node metastasis
LLNM: Lateral cervical lymph node metastasis
ETE: Extrathyroidal extension
HT: Hashimoto’ s thyroiditis
ATA: American Thyroid Association
AJCC: American Joint Committee on Cancer
